# Supplementary material for: A Mississippian (early Carboniferous) tetrapod showing early diversification of the hindlimbs
Source: Commun Biol. 2022 Apr 14;5:283. doi: 10.1038/s42003-022-03199-x (PMC9010477; doi:10.1038/s42003-022-03199-x)
Supplement: Supplementary file 2 — Supplementary Information [file 42003_2022_3199_MOESM2_ESM.pdf]

Supplementary Note 1: Characters

List of characters. The list provides the character formulation and character-state delimitations used in the present study.

1. Nostril outline morphology: slit-like (0), round (1), elliptical and transversely wide (2), or elliptical and anteroposteriorly elongate (3).
2. Nostril maximum diameter: less wide than premaxilla (0) or almost as wide as premaxilla (1) in dorsal aspect.
3. Premaxilla alary process: absent (0) or present (1).
4. Width of conjoined premaxillae: more than (0) or less than (1) two-thirds of the width of the skull table measured as the widest distance between its lateral margins.
5. Anterodorsally to posteroventrally oblique orientation of anterior surface of premaxilla, resulting in mouth opening subterminally: absent (0) or present (1).
6. Shelf-like contact between palatal surfaces of premaxilla and maxilla occurring mesial to marginal tooth row and extending mesially for at least twice the width of such a row: absent (0) or present (1).
7. Contribution of premaxilla to choanal margin: broad contact (0), point-like contact (1), or exclusion from choanal margin by vomer (2).
8. Palatal process on premaxilla: absent (0) or present (1).
9. Septomaxilla forming detached ossification inside nostril: absent (0) or present (1).
10. Septomaxilla contact with nasal: present (0) or absent (1).
11. Nasal length: more than (0) or less than (1) one-third as long as frontals at skull midline.
12. Parietal/nasal length ratio: less than (0) or more than 1.45 (1).
13. Nasal-frontal sutural course: smooth (0) or interdigitating (1).
14. Nasal contribution to narial margin: absent (0) or present (1).
15. Prefrontal length: less than (0) or more than (1) three times longer than wide.
16. Prefrontal-premaxilla suture: absent (0) or present (1).
17. Stout, lateral outgrowth of prefrontal: absent (0) or present (1).
18. Contribution of prefrontal to nostril margin: absent (0) or present (1).
19. Prefrontal-maxilla suture: absent (0) or present (1).
20. Prefrontal contribution to orbit anteromesial margin: more than (0) or less than (1) half of this margin.
21. Prefrontal extending in front of anterior margin of frontal: present (0) absent (1).
22. Prefrontal-postfrontal contact: broad (0), point-like (1), or absent (2).
23. Position of prefrontal-postfrontal suture relative to frontal: posterior to (0), at the same level as (1), or anterior to (2) frontal mid-length.
24. Position of prefrontal-postfrontal suture relative to orbit: anterior half (0) or middle to posterior half of orbit length (1).
25. Lacrimal relationship relative to prefrontal and jugal: lacrimal preventing (0) or not preventing (1) contact between prefrontal and jugal.
26. Anteriorly directed, V-shaped emargination along posterior margin of lacrimal: absent (0) or present (1).
27. Total length of lacrimal: less than (0) or more than (1) two and a quarter times its maximum pre-orbital length.
28. Lacrimal contribution to narial margin: present (0) or absent (1).
29. Location of dorsalmost point of maxilla in lateral aspect: in anterior third of maxilla length (0) or approximately at its mid-length (1).
30. Position of posterior extremity of maxilla: extending posterior to orbit’s posterior margin (0) or occurring anterior to this margin (1).

31. Frontal length: shorter (0), longer (1), or approximately equal in length (2) to parietals.
32. Anterior margin of frontal deeply wedged into nasal posterolateral margin of nasal: absent (0) or present (1).
33. Parietal-tabular suture: absent (0) or present (1).
34. Parietal-postorbital suture: absent (0) or present (1).
35. Position of anterior margin of parietal: anterior to (0), level with (1), or posterior to (2) orbit mid-length.
36. Parietal length: more than (0) or less than (1) two and a half times as long as wide.
37. Parietal-squamosal suture partly extending onto dorsal surface of skull table: absent (0) or present (1).
38. Parietal-frontal sutural course: smooth (0) or interdigitating (1).
39. Parietal-postparietal sutural course: smooth (0) or interdigitating (1).
40. Parietal lateral lappets: absent (0) or present (1).
41. Postparietal set: paired (0) or unpaired (1).
42. Postparietal length: less than (0) or more than (1) four times wider than long.
43. Postparietal median lappets: absent (0) or present (1).
44. Postparietal-exoccipital suture: absent (0) or present (1).
45. Postparietal occurring entirely on occipital surface of skull: absent (0) or present (1).
46. Posteromedial extensions of occipital flanges of postparietals projecting posteroventrally forming posteriorly directed process: absent (0) or present (1).
47. Nasal size (exposed surface) relative to postparietals: nasals larger or approximately identical in size to (0) or smaller than postparietals (1).
48. Midline ‘peak’ projecting posteriorly from conjoined posterior margin of postparietals: absent (0) or present (1).
49. Postfrontal posterior margin approximately aligned transversely with jugal posterior margin: absent (0) or present (1).
50. Postfrontal outline approximately chevron-shaped: absent (0) or present (1).
51. Separately ossified intertemporal: present (0) or absent (1).
52. Intertemporal contact with cheek region: not interdigitating (0) or interdigitating (1).
53. Intertemporal-squamosal suture: absent (0) or present (1).
54. Intertemporal shaped like a small, sub-quadrangular bone less than half as extensive (exposed surface) as supratemporal: absent (0) or present (1).
55. Supratemporal bordering entire edge of dorsalmost part of temporal notch: absent (0) or present (1).
56. Supratemporal strap-like and at least three times as long as wide: absent (0) or present (1).
57. Supratemporal-squamosal sutural course: smooth (0) or interdigitating (1).
58. Supratemporal margin contributes to squamosal embayment: absent (0) or present (1).
59. Supratemporal-intertemporal margin sutural course: irregular (0) or smoothly convex (1).
60. Supratemporal forming small, narrow, splint-like element: absent (0) or present (1).
61. Separately ossified tabular: present (0) or absent (1).
62. Morphology of posterolateral process (extension) of tabular: absent (0), spike-like unornamented ‘horn’ (1), elongate, recurved, unornamented, and dorsoventrally flattened blade (2), wide, subrectangular, unornamented, and plate-like bony sheet (3), conical extension of unornamented portion of posterolateral corner of tabular (4), or small, quadrangular, ornamented process (5).
63. Rounded, button-like posterior process of tabular occurring ventral to tabular ornamented surface: absent (0) or present (1).
64. Tabular-squamosal suture: present (0) or absent (1).
65. Width of conjoined parietals: smaller than (0) or greater than (1) the distance between the skull table posterior margin and the orbit posterior margin, measured along skull midline.

66. Tabular positioned entirely on occipital surface: absent (0) or present (1).
67. Tabular width relative to supratemporal: narrower (0) or broader (1) than supratemporal in dorsal view.
68. Postorbital outline: irregularly polygonal (0) or broadly crescentric, narrowing to a posterior point (1).
69. Postorbital width relative to orbit: less wide than or approximately equal in width to orbit (0) or wider (1) than orbit.
70. Postorbital width at least one-fourth of the width of the skull roof at the same transverse level: absent (0) or present (1).
71. Anteriormost part of the postorbital mesial margin with sigmoid profile in dorsal or lateral aspect: absent (0) or present (1).
72. Postorbital proportions: not broader than long (0) or broader than long (1).
73. Postorbital proportions in its anterolateroventral portion: absence (0) or presence (1) of drawn out, acutely triangular process bordering posterior orbit margin.
74. Postorbital-supratemporal contact: present (0) or absent (1).
75. Quadratojugal size relative to squamosal: quadratojugal larger than (0) or smaller than squamosal (1).
76. Quadratojugal in the form of anteroposteriorly elongate and dorsoventrally narrow splinter of bone: absent (0) or present (1).
77. Spatial relationships between quadratojugal and jugal (lateral aspect of suture, if present): quadratojugal underlying jugal (0), jugal-quadratojugal suture oriented approximately vertically (1), or jugal underlying quadratojugal (2).
78. Position of dorsalmost part of quadratojugal relative to maxilla: above (0) or below (1) highest point of maxilla.
79. Position of anteriormost part of squamosal relative to parietal mid-length: posterior to (0) or anterior to (1) parietal mid-length.
80. Broad, concave embayment of squamosal: absent (0) or present (1).
81. Location of squamosal-supratemporal suture: at apex of temporal embayment (0), dorsal to apex, (1) or ventral to apex (2).
82. Unornamented otic flange of squamosal: absent (0), approximately uniformly narrow dorsoventrally (1), narrow dorsally and widening ventrally (2), or narrow ventrally and widening dorsally (3).
83. Jugal contribution to skull table ventral margin: absent (0) or present (1).
84. Jugal-pterygoid contact: absent (0) or present (1).
85. Depth of jugal ventral to orbit: greater than (0) or smaller than (1) half of anteroposterior orbit diameter.
86. V-shaped indentation of orbital margin of jugal: absent (0) or present (1).
87. Position of anterior extremity of jugal relative to anterior margin of orbit: anterior extremity of jugal not extending (0) or extending (1) anterior to anterior margin of orbit.
88. Quadrate dorsal process: absent (0) or present (1).
89. Preopercular: present (0) or absent (1).
90. Size of nostril relative to size of choana: nostril less than (0) or equal to or greater than (1) 50 percent of the size of the choana.
91. Naris height in lateral projection: greater than (0) or equal to or less than (1) the distance between the naris ventral rim and the upper jaw margin.
92. Internarial fenestra: absent (0) or present (1).
93. Interorbital distance: greater than (0), smaller than (1), or sub-equal to (2) half of the skull table width measured as the widest distance between its lateral margins.
94. Interorbital distance: greater than (0), smaller than (1), or sub-equal to (2) the maximum orbit diameter.

95. Angular profile at the anteroventral corner of the orbit: absent (0) or present (1).
96. Orbit proportions: orbit not deeper than long (0) or deeper than long (1).
97. Orbit anteroposterior diameter: shorter than (0), longer than (1), or sub-equal to (2) the distance between the orbit posterior margin and the suspensorium anterodorsal margin.
98. Elliptical orbit with long axis anterodorsally to posteroventrally orientated: absent (0) or present (1).
99. Position of midpoint of maximum anteroposterior orbit diameter: closer to anterior extremity of the snout than to the posterior extremity of the skull (0), situated approximately at skull mid-length (1), or closer to the posterior extremity of the skull than to anterior extremity of the snout (2).
100. Antorbital vacuities confluent with orbits: absent (0) or present (1).
101. Size of greatest diameter of pineal foramen: less than (0) or more than (1) 33% or greater than the anteroposterior length of the parietal suture.
102. Pineal foramen position: posterior to (0), at the level of (1), or anterior to (2) interparietal suture mid length, or absent (3).
103. Posttemporal fenestra position: occurring at, delimited dorsally by the skull table, not bordered laterally, and floored by a dorsolateral extension of the opisthotic (0), occurring near the dorsolateral corner of the occiput, delimited dorsally by the occipital flanges of the tabular and postparietal, and bordered laterally as well as ventrally by dorsolateral extensions of the opisthotic meeting the ventromedial flange of the tabular (1), small fossa occurring near ventrolateral corner of the occiput, bordered laterally by ventromedial flange of the tabular, delimited dorsally by the dorsal portion of the lateral margin of the supraoccipital-opisthotic complex, and floored by a lateral extension of the opisthotic (2), or absent altogether (3).
104. Squamosal contribution to posttemporal fenestra: absent (0) or present (1).
105. Distance between quadrate and anterior margin of temporal embayment: equal to (0), less than (1), or greater than (2) the maximum width of the orbit.
106. Shallow, subvertical temporal embayment: absent (0) or present (1).
107. Postorbital region of skull table abbreviated and at least one-third wider than long: absent (0) or present (1).
108. Skull general proportions: skull longer than broad (0), as broad as long (1), or broader than long (2).
109. Embayments of skull lateral margins: absent (0) or present (1).
110. proportions of preorbital region of the skull: less than twice as wide as long (0) or at least twice as wide as long (1).
111. Dermal skull ornament: regular with starburst patterns at areas of growth (0), irregular and deep (1), irregular and shallow (2), or partially or fully absent (3).
112. Expression of lateral line system on skull table: totally enclosed (0), mostly enclosed with short sections in grooves (1), mostly in grooves with short sections enclosed (2), entirely in grooves (3), or absent (4).
113. Expression of lateral line system on lower jaw: totally enclosed (0), mostly enclosed with short sections in grooves (1), mostly in grooves with short sections enclosed (2), entirely in grooves (3), or absent (4).
114. Lower temporal fenestra delimited by postorbital, squamosal, and jugal: absent (0) or present (1).
115. Ventral, exposed surface of vomers (excluding any areas of overlap with surrounding bones) narrow, elongate and strip-like, without extensions anterolateral and posterolateral to the choana, and two and a half to three times longer than wide: absent (0) or present (1).
116. Vomerine fangs comparable in size to, or larger than, marginal teeth of either the premaxilla or maxilla: present (0) or absent (1).

117. Vomerine fangs noticeably smaller than other palatal fangs: absent (0) or present (1).
118. Vomer small teeth (denticles) forming continuous shagreen or discrete, patches, the basal diameter and/or height of which is less than 30% of that of adjacent marginal premaxillary or maxillary teeth and any remaining vomer teeth (if present): absent (0) or present (1).
119. Vomer contribution to interpterygoid vacuities: vomer excluded from (0) or contributing to (1) interpterygoid vacuities.
120. Vomer-maxilla suture anterior to the choana: absent (0) or present (1).
121. Toothed lateral crest on palatal surface of vomer: present (0) or absent (1).
122. Transversely orientated, anterior crest on palatal surface of vomer: present (0) or absent (1).
123. Amount of separation between vomers by intervening pterygoids: vomers separated for more than (0) or less than (1) half of their length, or not separated (2).
124. Palatine fangs fangs comparable in size to or larger than marginal teeth (premaxillary or maxillary): present (0) or absent (1).
125. Palatine small teeth (denticles) forming continuous shagreen or discrete patches, the basal diameter and/or height of which is less than 30% of that of adjacent marginal teeth (maxillary) and remaining vomer teeth (if present): absent (0) or present (1).
126. Palatine row of 3 or more teeth comparable in size to, or greater than marginal maxillary teeth and parallel to these: present (0) or absent (1).
127. Separately ossified ectopterygoid: present (0) or absent (1).
128. Ectopterygoid fangs comparable in size to or larger than marginal premaxillary or maxillary teeth and any remaining ectopterygoid teeth: present (0) or absent (1).
129. Ectopterygoid small teeth (denticles) forming continuous shagreen or discrete patches, the basal diameter and/or height of which is less than 30% of that of adjacent marginal premaxillary or maxillary teeth and any remaining ectopterygoid teeth (if present): absent (0) or present (1).
130. Ectopterygoid proportions: longer than or as long as palatine (0) or shorter than palatine (1).
131. Ectopterygoid row of three or more teeth comparable in size to, or greater than, marginal maxillary teeth and parallel to these: present (0) or absent (1).
132. Ectopterygoid contribution to adductor fossa: absent (0) or present (1).
133. Mesial exposure of palatal surface of palatine and ectopterygoid: narrow, smaller than or comparable to basal diameter of marginal teeth (0) or broad, larger than basal diameter of marginal teeth (1).
134. Single row of large teeth on the anterior process of the palatal ramus of the pterygoid: absent (0) or present (1).
135. Dentition pattern on transverse flange of pterygoid: absence of transverse tooth row and/or occurrence of shagreen (0), row of large teeth (1), or row of small teeth (2).
136. Transverse pterygoid flange: absent (0), present as an incipient downturning (torus transiliens) (1), or present as a distinct ridge (2).
137. Posterolateral flange of pterygoid: absent (0) or present (1).
138. Pterygoids sutured with each other: absent (0) or present (1).
139. Distinct, mesially directed process of pterygoid corpus for basipterygoid articulation: absent (0) or present (1).
140. Vomer-palatine contact: present (0) or absent, due to intervening pterygoid (1).
141. Secondary palatal shelf of palatine and ectopterygoid: absent (0) or present (1).
142. Interpterygoid vacuities: present (0) or absent (1).
143. Extension of interpterygoid vacuities: vacuities not occupying (0) or occupying (1) at least half of the palatal width.
144. Profile of interpterygoid vacuities: vacuities not concave (0) or concave (1) along their entire margins.

145. Proportions of interpterygoid vacuities: conjoined vacuities not broader than long (0) or broader than long (1).
146. Anterior palatal vacuity: present and single (0), present and double (1), or absent (2).
147. Ossified supraoccipital: absent (0) or present (1).
148. Exoccipitals enlarged, about as broad as high and forming stout occipital condyles: absent (0) or present (1).
149. Basioccipital notochordal: present (0) or absent (1).
150. Overall morphology of basioccipital articulation: not convex (0) or broadly convex (1).
151. Opisthotic forming a thickened plate fused together with the supraoccipital, preventing the exoccipitals from contacting the skull table: absent (0) or present (1).
152. Occiput orientation: nearly vertical (0) or sloping anterodorsally (1).
153. Elongate, strut-like cultriform process of parasphenoid: absent (0) or present (1).
154. Anterior, triangular, wedge-like, more or less distinct process immediately anterior to the level of the basipterygoid processes on the ventral surface of the parasphenoid: absent (0) or present (1).
155. Posterolaterally orientated, paired ventral thickenings (ridges ending in basal tubera) on parasphenoid: absent (0) or present (1).
156. Broad and elongate posterolateral processes of parasphenoid: absent (0), present and less than half as wide as parasphenoid ventral plate (1), or at least half as wide as parasphenoid ventral plate (2).
157. Single median depression on ventral surface of parasphenoid: absent (0) or present (1).
158. Paired lateral depressions on ventral surface of parasphenoid: absent (0) or present (1).
159. Ventral cranial fissure: sutured and traceable (0) or not traceable (1).
160. Dentition pattern on parasphenoid cultriform process: shagreen (0), patch of denticles (1), radiating ridges and denticle rows (2), or smooth (3).
161. Parasphenoid-vomer contact: present (0) or absent (1).
162. Lower jaw dimensions: shorter than (0), subequal to (1), or deeper than (2) the skull in lateral view.
163. Jaw articulation position: posterior to (0), level with (1), or anterior to (2) the occiput.
164.) Parasymphysial plate: present (0) or absent (1).
165. Paired fangs comparable in size with or greater than dentary teeth on parasymphysial plate: absent (0) or present (1).
166. Parasymphysial plate without (0) or with (1) anteroposterior tooth row orientated subparallel to the marginal dentary teeth and the basal diameter and/or height of which is 30% or greater than that of the marginal teeth and twice or more that of denticles, if present.
167. Parasymphysial plate with (0) or without (1) small teeth (denticles) forming continuous shagreen or discrete patches and the basal diameter and/or height of which is less than 30% of that of the adjacent marginal dentary teeth.
168. Accessory tooth rows on the dentary: present (0) or absent (1).
169. Dentary anterior fangs generally comparable in size with, or greater than, other dentary teeth and lying close to symphysial region and usually mesial to marginal dentary teeth: present (0) or absent (1).
170. Sigmoid profile of upper margin of dentary in lateral aspect: absent (0) or present (1).
171. Posteriormost extension of splenial mesial lamina closer to anterior margin of adductor fossa than to anterior extremity of jaw, when the lower jaw ramus is observed in mesial aspect and in anatomical connection: absent (0) or present (1).
172. Splenial-anterior coronoid suture: absent (0) or present (1).
173. Splenial-middle coronoid suture: absent (0) or present (1).
174. Separately ossified postsplenial: present (0) or absent (1).
175. Postsplenial mesial lamina: absent (0) or present (1).

176. Angular mesial lamina: absent (0) or present (1).
177. Position of posterior extremity of angular in lateral aspect: angular not reaching (0) or reaching (1) posterior extremity of lower jaw.
178. Lateral exposure of surangular much smaller than lateral exposure of angular: absent (0) or present (1).
179. Prearticular-splenial suture: present (0) or absent (1).
180. Denticulated field on prearticular: defined edges (0), scattered patches (1), or absent (2).
181. Separately ossified anterior coronoid: present (0) or absent (1).
182. Anterior coronoid fangs comparable in size to or larger than the marginal dentary teeth: present (0) or absent (1).
183. Anterior coronoid with (0) or without (1) small teeth (denticles) forming continuous shagreen or discrete patches and the basal diameter and/or height of which is less than 30% of that of the adjacent marginal dentary teeth.
184. Tooth row on the anterior coronoid: present (0) or absent (1).
185. Separately ossified middle coronoid: present (0) or absent (1).
186. Middle coronoid fangs comparable in size to or larger than the marginal dentary teeth: present (0) or absent (1).
187. Middle coronoid with (0) or without (1) small teeth (denticles) forming continuous shagreen or discrete patches and the basal diameter and/or height of which is less than 30% of that of the adjacent marginal dentary teeth.
188. Middle coronoid with (0) or without (1) anteroposterior tooth row orientated subparallel to marginal dentary teeth and the basal diameter and/or height of which is 30% or greater than that of marginal teeth and twice or more that of the denticles, if present.
189. Posterior coronoid with (0) or without (1) small teeth (denticles) forming continuous shagreen or discrete patches and the basal diameter and/or height of which is less than 30% of that of the adjacent marginal dentary teeth.
190. Posterior coronoid with (0) or without (1) anteroposterior tooth row orientated subparallel to the marginal dentary teeth and the basal diameter and/or height of which is 30% or greater than that of marginal teeth and twice or more that of the denticles, if present.
191. Posterodorsal process of posterior coronoid: absent (0) or present (1).
192. Exposure of posterior coronoid in lateral view: absent (0) or present (1).
193. Contribution of posterodorsal process of posterior coronoid to tallest point of lateral margin of adductor fossa: absent (0) or present (1).
194. Adductor fossa orientation: facing dorsally (0) or mesially (1).
195. Posterior Meckelian fenestra between prearticular and angular: absent (0), present and small (1), or present and large (2).
196. Anterior Meckelian fenestra between splenial, postsplenial, and prearticular: absent (0), present and small (1), or present and large (2).
197. Single, large elongate Meckelian fenestra resulting in narrow mesial exposure of splenial: absent (0) or present (1).
198. Anterior elongate fenestra between ventromedial anterior lamina of dentary and splenial: absent (0) or present (1).
199. Teeth in-curved in anterior aspect: absent (0) or present (1).
200. Conspicuous peak involving one or more premaxillary teeth: absent (0) or present (1).
201. Conspicuous peak involving one or more anterior maxillary teeth: absent (0) or present (1).
202. Size of dentary teeth relative to that of maxillary teeth: dentary teeth not larger (0) or larger (1) than maxillary teeth.
203. Marginal tooth crowns chisel-tipped: absent (0) or present (1).
204. Number of maxillary teeth: greater than 40 (0), between 30 and 40 (1), or less than 30 (2).
205. Number of premaxillary teeth: greater than 4 (0), equal to 4 (1), or less than 4 (2).

206. Postbranchial lamina of cleithrum: present (0) or absent (1).
207. Cleithrum distal end marked from a narrow stem by a notch or process, or a decrease in thickness (0) or tapering (1).
208. Cross-section of cleithrum stem: flattened oval (0), irregular outline (1), or with a single concave face (2).
209. Anterior contact between clavicles: present (0) or absent (1).
210. Interclavicular stem: absent (0), slightly elongated and subtriangular with blunt posterior end (1), elongate and rod-like and with broadened anterior portion (2), elongate and rod-like and with broadened posterior portion (3), elongate and rod-like and with mid-length expansion along shaft (4), elongate and rod-like but without expansion along shaft and shaft with more or less posteromedially converging lateral margins (5).
211. Interclavicle proportions: not wider than long (excluding parasternal process, if present) (0) or wider than long (1).
212. Interclavicle shape: rhomboidal with posterior part longer than (0) or shorter than (1) anterior part.
213. Transversely elongate grooves and ridges on central part of interclavicle ventral surface: absent (0) or present (1).
214. Separate scapular ossification: absent (0) or present (1).
215. Glenoid location: subterminal (0) or non-subterminal (1).
216. Separately ossified anocleithrum: present (0) or absent (1).
217. Morphology of latissimus dorsi process: part of a ridge (0), distinct but low (1), or spike-like (2).
218. Latissimus dorsi process position: offset anteriorly relative to the ectepicondyle (0) or aligned with the latter (1).
219. Distinct supinator process projecting anteriorly: absent (0) or present (1).
220. Sharp-edged, ventral humeral ridge: present (0) or absent (1).
221. Ectepicondyle foramen: present (0) or absent (1).
222. Distinct ectepicondyle ridge: present (0) or absent (1).
223. Position of the distal extremity of the ectepicondyle ridge: aligned with the ulnar condyle (0), between the ulnar and radial condyles (1), or aligned with the radial condyle (2).
224. Humerus waisted shaft: absent (0) or present (1).
225. Humerus torsion: little torsion, planes of flattening of proximal and distal ends lying approximately flush with one another (0) or torsion offsetting proximal and distal ends by 60 degrees or more (1).
226. Position of radial condyle: terminal (0) or ventral (1).
227. Humerus length: less than (0) or more than (1) three times the maximum width of its distal end.
228. Width of entepicondyle: greater than (0) or smaller than (1) than half humeral length.
229. Portion of humeral shaft length proximal to entepicondyle: smaller than (0) or greater than (1) humerus head width.
230. Accessory foramina on the humerus: present (0) or absent (1).
231. Humerus length: greater than (0) or less than (1) the combined length of two and a half mid-trunk vertebrae.
232. Process ‘2’ on the humerus: absent (0) or present (1).
233. Entepicondylar foramen: present (0) or absent (1).
234. Radius length relative to ulna length: radius longer than (0), as long as (1), or shorter than (2) the ulna.
235. Olecranon process: absent (0) or present (1).

236. Morphology of dorsal iliac process profile: process absent (0), flared (1), subrectangular (2), stub-like (3), broadly semicircular (4), 5 drawn out and finger-like (5), or broadly subelliptical (6).
237. Posterior iliac process with dorsal and ventral margins abbreviated and strongly converging posteriorly: absent (0) or present (1).
238. Transverse pelvic ridge: absent (0), present as a sharp crest (1), or present as a subhorizontal protruding shelf (2)
239. Morphology of posterior iliac process: process absent or stout and foreshortened (0), with a slender, rectangular, and blade-like profile (1), or blunt and subrectangular (2), or sinuous with stepped proximal portion of ventral margin (3).
240. Number of pubic obturator foramina: multiple (0), single (1), or absent (2).
241. Internal trochanter raised as a distinct protuberance: absent (0) or present (1).
242. Internal trochanter separated from the general surface of the femur shaft by a distinct, trough-like space: absent (0) or present (1).
243. Fourth trochanter of the femur with a distinct rugose area: absent (0) or present (1).
244. Proximal end of the femur adductor crest reaching midshaft length: absent (0) or present (1).
245. Femur length relative to humerus length: femur shorter than (0), as long as (1), or longer than the humerus (2).
246. Outline of the tibia medial margin shaped like a distinct, subsemicircular embayment contributing to interepipodial space and the diameter of which is less than one-third of the bone length: absent (0) or present (1).
247. Flange along posterior edge of tibia: absent (0) or present (1).
248. Fibula waisted: absent (0) or present (1).
249. Ridge near the posterior edge of the flexor surface of the fibula: absent (0) or present (1).
250. Rows of tubercles near the posterior edge of the flexor surface of the fibula: absent (0) or present (1).
251. L-shaped proximal tarsal element: absent (0) or present (1).
252. Flattened distal extremities of cervical ribs: present (0) or absent (1).
253. Trunk rib shaft: mostly straight (0) or ventrally curved (1) in at least part of the trunk.
254. Broad rectangular flanges on trunk ribs: absent (0) or present (1) in at least some trunk ribs.
255. Greatest trunk rib length: ribs shorter than combined height of neural arch and centrum (0) up to two and a half times this height (1), or more than (2) two and a half times this height.
256. Number of sacral vertebrae: one (0) or two (1).
257. Number of presacral vertebrae: 30 (0), 30–38 (1), more than 38 (2), 28–30 (3), or fewer than 28 (4).
258. Axis arch fusion with axis (pleuro)centrum: absent (0) or present (1).
259. Atlantal pleurocentrum preventing contact between the atlantal and axial intercentra (0) or not preventing contact (1).
260. Relationship of atlantal pleurocentrum to axial intercentrum: atlantal pleurocentrum in contact with or narrowly separated from anterior surface of axial intercentrum (0); atlantal pleurocentrum articulating with or fused to the dorsal surface of the axial intercentrum (1).
261. Anteriorly directed, mid-ventral process of axial intercentrum: absent (0) or present (1).
262. Absence (0) or presence (1) of ossified pleurocentra.
263. Trunk pleurocentra fused midventrally: absent (0) or present (1).
264. Trunk pleurocentra fused middorsally: absent (0) or present (1).
265. Difference in height between trunk pleurocentra and intercentra: more than 25% (0) or roughly equal in height (1).
266. Distinct convex lateral surfaces of neural arches, conferring to them a ‘swollen’ appearance: absent (0) or present (1).
267. Neural arches of trunk vertebrae fused to centra: absent (0) or present (1).

268. Trunk intercentra fused mid-dorsally: absent (0) or present (1).
269. Tallest ossified part of neural arch in posterior trunk vertebrae aligned vertically with the posterior half of vertebral centrum: absent (0) or present (1).
270. Postzygapophyses on trunk vertebrae: absent (0) or present (1).
271. Height of ossified portion of neural arch in mid-trunk vertebrae: greater than (0) or smaller than (1) the distance between pre- and postzygapophyses.
272. Sacral centra distinguishable by size and shape from pre- and post-sacral centra: absent (0) or present (1).
273. Manus digit number: five (0) or four (1).
274. Astragalus: tibiale, intermedium, and proximal centrale not fused to form an astragalus (0) or fused to form an astragalus (1).
275. Ventral scale morphology: tapered and elongate (0) or ovoid (1).
276. Inner ear configuration: non-amniote-type inner ear, with small cochlear recess forming small extension of the vestibule (0) or amniote-type inner ear, with large cochlear recess of mostly triangular shape in lateral view (1).
277. Position of cochlear recess relative to vestibule: posterior (0), posteroventral (1), or ventral (2).
278. Distinctly developed subarcuate fossa in the braincase bone(s) housing flocculus of cerebellum: absent (0) or present (1.
279. Otic tube (=otic canal): absent (0) or present (1).
280. Otic trough: absent (0) or present (1).
281. Course of semicircular canals: entire or posteromedial section of anterior semicircular canal and entire posterior semicircular canal with straight course (lying in horizontal plane and meeting at an approximately right angle in the region of the crus commune) (0) or semicircular canals with distinct arcuate course (1).
282. Posterolateral corner of skull table: formed entirely by tabular (0), formed entirely or nearly entirely by supratemporal (1) or with subequal contributions from supratemporal and tabular (2).
283. Position of jaw articulation: approximately at the same level as the dental occlusal plane (0) or below level of dental occlusal plane (1).
284. Labial parapet: absent (0); present and low, with parapet never projecting higher than the bases of the cheek teeth (1), or present and tall, with parapet as tall as or taller than the occlusal surface of the teeth near the posterior end of the tooth row (2).
285. Degree of molarization of the largest preserved mid-series cheek teeth: absence of molarization (0), low degree of molarization in the development of labial and lingual cusps or shoulders and transverse expansion of crowns (1), or high degree of molarization with well-developed labial and lingual cusps or shoulders (ratio of anteroposterior length to mediolateral width and dorsoventral height to mediolateral width in the mid-series cheek teeth both less than 0.50) (2).
286. Processes of the atlantal neural spines: atlantal neural spines with large, posterodorsally directed processes (0) or atlantal neural spines with small epipophyses (1).
287. Shape of unguals of manus and pes: long, narrow, recurved, and pointed (0) or hoof-like (short, flattened, bluntly pointed to rounded, and with the dorsal and ventral surfaces meeting along the lateral and distal margins in a narrow edge) (1).
288. Keratinous sheath or claw of manus and pes unguals: absent (0) or present (1).
289. Iliac process (posterior) obliqueness (greater axis): absent or imperceptible (0) or present and conspicuous (1).
290. Ventrolateral (jugal) ramus of postorbital: absent or indistinct (0), incipient (1), or well-developed and elongate (2).

291. Length of postorbital region of jugal: more than (0) or less than (1) one-third of the length of the postorbital cheek region.
292. Elongation of 4th metatarsal: less elongate than all other metatarsals, less than 30% than 3rd metatarsal (0) or at least 30% or more than 3rd metatarsal (1).
293. Proportions of 4th metatarsal: not distinctly stouter/wider (0) or distinctly stouter/wider (1) than all metatarsals.
294. Profile of the posterior margin of the pelvis: sinuous profile under iliac neck, with large convexity above ilium-ischium suture, and with abbreviated ischium (0), spatulate to narrow triangular ischium with broadly concave dorsal margin, and small convexity above ilium-ischium suture (1), straight, gently sloping dorsal margin of ischium (2), gently or poorly pronounced 'stepped' profile around ilium-ischium suture, elongate ischium with broadly concave dorsal margin (3), or deeply angular dorsal margin of ischium, ischium with stout plate-like shape (4).
