# Supplementary material for: A Mississippian (early Carboniferous) tetrapod showing early diversification of the hindlimbs
Source: Commun Biol. 2022 Apr 14;5:283. doi: 10.1038/s42003-022-03199-x (PMC9010477; doi:10.1038/s42003-022-03199-x)
Supplement: Supplementary file 3 — Description of Additional Supplementary Files [file 42003_2022_3199_MOESM3_ESM.pdf]

Description of Additional Supplementary Files

File Name: Supplementary Data 1

Description: Data matrix and list of characters
