# Supplementary material for: A Mississippian (early Carboniferous) tetrapod showing early diversification of the hindlimbs
Source: Commun Biol. 2022 Apr 14;5:283. doi: 10.1038/s42003-022-03199-x (PMC9010477; doi:10.1038/s42003-022-03199-x)
Supplement: Supplementary file 4 — Supplementary Data 1 [file 42003_2022_3199_MOESM4_ESM.docx]

Supplementary Data 1: Data matrix

**Taxon-character matrix in Nexus format.** The text enclosed between the sets of double lines, beginning with ‘#NEXUS’ and ending with ‘END;’, is PAUP*-readable. Character-states within curly { } and round ( ) brackets denote, respectively, uncertain and polymorphic coding.

============================================================================

#NEXUS

BEGIN TAXA;

DIMENSIONS NTAX=55;

TAXLABELS

Anthracosaurus_russelli Archeria_crassidisca Ariekanerpeton_sigalovi Balanerpeton_woodi Baphetes_kirkbyi Bruktererpeton_fiebigi Caerorhachis_bairdi Calligenethlon_watsoni Captorhinus_aguti Carbonoherpeton_carrolli Chroniosaurus_dongusensis Crassigyrinus_scoticus Dendrysekos_helogenes Desmatodon_hesperis Diadectes_absitus Diadectes_sideropelicus Diasparactus_zenos Dimetrodon_grandis Discosauriscus_austriacus Eobaphetes_kansensis Pholiderpeton_attheyi Eoherpeton_watsoni Eothyris_parkeri Eucritta_melanolimnetes Gephyrostegus_bohemicus Greererpeton_burkemorani Karpinskiosaurus_secundus Labidosaurus_hamatus Leptoropha_talanophora Limnoscelis_paludis Makowskia_laticephala Megalocephalus_pachycephalus Microphon_exiguum Oedaleops_campi Orobates_pabsti Ossinodus_pueri Palaeoherpeton_decorum Paleothyris_acadiana Pederpes_finneyae Petrolacosaurus_kansensis Pholiderpeton_scutigerum Proterogyrinus_pancheni Proterogyrinus_scheelei Seymouria_baylorensis Seymouria_sanjuanensis Silvanerpeton_miripedes Solenodonsaurus_janenschi Spinarerpeton_brevicephalum Tseajaia_campi Utegenia_shpinari Varanops_brevirostris Westlothiana_lizziae Whatcheeria_deltae Eldeceeon_rolfei Termonerpeton_makrydactylus

;

END;

BEGIN CHARACTERS;

DIMENSIONS NCHAR=294;

FORMAT DATATYPE = STANDARD GAP = - MISSING = ? SYMBOLS = " 0 1 2 3 4 5 6";

MATRIX

Anthracosaurus_russelli ?0010000??001110000000000010001010210000001001010001000011000101101000000000011011001110110011002020023?201000244?11?0?011?0?100000100?00?000000020010100010101?110????110?10011?0?201??????0000??00100010022????????????????????????????????????????????????????????????????????????????0000????00???

Archeria_crassidisca 210100?0??000110000000001010011010210010001001010000000001100101001100001?000010111110101?0011001020023?000000{0 1}3?0????0???????0?0?000001010000000?00101000101013?00000111011101100020101010101110122000100102102110000011100102000001111021401111011200110101?20100001111001010100???????0000000101003

Ariekanerpeton_sigalovi 2101000001000100000001110000012010210000010?0000000010000100030110110101010001101210100?1000200020000210101101{1 2}33010010011001101101110021?00000002?0?0??011200131211???110?1?01110?2010101010110111?010000020112021001??1?01102110000110010?1?0?????20?1???010104????1100100010?001??????0000?0012100?

Balanerpeton_woodi 10100010??000100000001210000001000210000000?0000000101001100000100010000100000110200100011101100201002??101000044000?11011201100101110?01000001112?010??100000130?01???10001001100120101010101???10000000100010?100000?1??0111?1100001100110003?11?020010000000040?0010000000100101??????000000010100?

Baphetes_kirkbyi 1000001000001100100000000100001000210??0000000000001011011000011000000000000101023100100110012002021001020000003?000010011201100101110?0010001???200?010000001101?00101??0???0?????2??????????????????0000010?01????????20001020100001?00??{2 3}00???????00110?????04??????????0?????00??????0000????00???

Bruktererpeton_fiebigi 3?0???{0 1}???0001?0?0000?????101?1010210000010?0?00?000?000001000??000???????????????????????10??????2002???01000{2 3}??0?00?????20??011??110???1?00?????????????????????????????????????????????????????????0?0?0101{0 1}2?2???1??1101102001001110121?????110120010010102????001100000010??01??????0000000???00?

Caerorhachis_bairdi ??0???????00?1??????0001??0?00?000210110000?0?00?001?000??0000?000?100001000?0100??0????11?0?????0??0???2000??0??0?0110?111011001010?0?00100000002?0?????000101?100010010011001100000101010101100122000?0000??????????????????????????????1{3 4}011?11?1?001??10101?30?001100000010??00??????00000001??103

Calligenethlon_watsoni ????????????????????????????????11?10000001?0??1?00??0?0??10010?001?????????????????????????????????01???0????0{0 4}?????????????????????0???????1????????????????????????????????????????????????????????0?0?0?????1?11011????????1??0111?0121401111101?00100??101?1????111000(0 1)?101??0????????0????1????3

Captorhinus_aguti 110100200?001100000002??00101110?121100001001000011???000??110??0??0000011101010?01110101?00110000100211?00010344011?00011?1?11?????10120100000002101100001110101211???010???1?110021???1???11111101000110021???151000111101102111011110021000211111200100101021411101110110111101?121001?000101120003

Carbonoherpeton_carrolli ??????????????0?000?1???10??11?????????????????????????????????????0??0?0?0010?0??11?11?1?????01?1?0????????0?04?0????????????000?0???????????????????????????????0???????????????????????????????????0???02????????0????????????????1?00???????1111???????1101??????1111001010???????????000?????????

Chroniosaurus_dongusensis 30?10?????001100000002??1010002010210000000?0001001???00?110001100{0 1}10000101100110201101?11111200002002??00000014?0100100110010001?0110?01100000002??????011110131?0?????1??1??110?0??111010111???1????0010010???11001?????0111?1??00?1000??0001?0????1?1??0?10??40???111100101????1??????0000000120003

Crassigyrinus_scoticus 0000012011001100010001011000001000210??0001?01000100?0000110040100000000000000100100000011111110202000??200000113000000000001000000000?001000?0001??00??001010011200101100100001000101010101?????000100000010?01??000???110000{1 2}000000000000200??1111201110??102?1????0???0?0?00???0??????0000000100???

Dendrysekos_helogenes 101000?1100001000000001100000000002101100101000100010000110000110001000000000011120010?111112000001002??00000004?000111?11201100101?10?01010001102?110??10000013020??????0???01100??0???0????????11000000000010?10000001??011021100001100110013111?12001??00001?4????1000000010?1?1?????10000?00100001

Desmatodon_hesperis ??0??0?????????????????????????????????????????????????????????????????????????????????1??????????????20??????2????????????101???????112??1?1??????????????????????1???1111??1?110021???01111111100011001?121????????????????????????????????????????????????????1?1?111??1???????????????111?????????

Diadectes_absitus 001110001000110000000011000001101121000111001100011???0001?000011101000000100000?11010011110200010201220111000144011?0001121010101111102?10111???2?01110001110131211???1111??1?110021???1????111110011001012110012100111?011102111000100021612011010200100101011411111111110010?01?1011102122111101004

Diadectes_sideropelicus ??011000??00110000000011000011100121000110101100001????001?000011100000000101100?1100001100000000020112011100014?011?0?01121000101111102??011????2001110001010131????????1????????????????????????????0?1?121????2?????????????????????????????????????????????1?1111????????????1???????2122111?01004

Diasparactus_zenos ??????0????????????????????????????????????????????????????????????????????????????????0???????????0???????0?????0????????2?????????????????1?????????????????????1????101?????????2??????????1???001100??12110002?0011111111001110101000206120111?0100100?01?014????111?11?1101?1????????122?111?????

Dimetrodon_grandis (1 3)101(0 1)00?000001(0 1)0000002??00?101101121?0111010110?001????10??000011011000010????10?01?00111110200000201120?01000?44111?000112111011110102211000100021011111?????13??11???10111?1?1110211111???1111110000011002211?15000011?111102?1101010002101?01111120010011102141000111?01?11?1?1?????1?2?00101120004

Discosauriscus_austriacus 2101000001000100000001110000012010210000010?01000000100001100301101100010100001112101001100011002000021000110103301001001100110110111002110001???2?0?0??011200131211???11011101110020101010101111111010000020112021001001001102110000110010612011000200100?0001040???111010001000?1??????0000000121003

Eobaphetes_kansensis ???1???0????1100000010011011?01010210000001?0?0?000010?0011001001010000001001?001?01?1101???100?0?2000???0100013?0????????????00??????????????????1???????????????01???110010011001201?101?10100?122000???0??????????????????????????????????????????????????????????????????????????????0000?????????

Pholiderpeton_attheyi ??010?{1 2}0??001100000010001011001010210000001?0111000100000110010100000100100010101111111011101100002001??201000133011?0000011?100000100?00100000002??????0000101?1001???1100100110012010101010100?122000011102?0???100???????????????????????????0?0?????????1020?????111?011010??00??????0000000?00???

Eoherpeton_watsoni 100100????0011000000001?1010011010210000000?01010000?00000100401001?00000?0000?00100101?11?020?00020023?10000024401????????0?000?000?0?00?000?????????10?010101??2?????1?011101100000101010101?0?1?10?0000020????????01?11001010000011?1???401{1 3}1???1?00110??102??????1100000010??????????0000????00??3

Eothyris_parkeri 31?11?????001100000002??0000?0101121?11100101101011????00??000?100?1000010110?00?00?100?1?102100202011?0?00000?441????????????????????22??0???????1???11??????????1??????0??????10????????????111??????010?2{1 2}?????100????????????????????????????????????????????????????????????????????1?00????20???

Eucritta_melanolimnetes ??000??0??001100000000010100?020002100000?0?000000010110110000?1000100010000??002{1 2 3}1?100?11?01110100000??0000010??0?0010011101100??0?10?0?10001??????????0010101?1?0??????????????????????????????????????002??01110000?1??0?10?0000001?0011{2 4}0?0?????2001???00010??????????????????0??????0000?0010?001

Gephyrostegus_bohemicus 300100?0??000100000002??0010011110210000010?010?0000?0000?1005?10001000?10000010?100100011001100202002??2010002440100100???01100111110010100000002?0?0??01?2??13?2?1???1001110000002000100010111010010000000010212000011?10110200100111002110{0 1}11??0120011010102?4000011000000101011??????0000000120113

Greererpeton_burkemorani 10000110??110011011000000001001{0 1}0120011000000011001???001?0000100000110000001000000000001100100000100010100000022001?0001110?000000010?00100000001001010100001131?001011001100010002001001010111?0001001010000010001000111001010000001000210000111112001000100102000010000000100001??????0000000100001

Karpinskiosaurus_secundus 2101000001011100000000110000000010210010000?010000001010010000010011000111000111{0 1}200100?10011000000002??00020114401001001100110?101110021?00000002?0?0??011200131201???110???0111?02??????????11111?011000020????????????????????????????????????????????????????????????????????????????0000????20???

Labidosaurus_hamatus 3111100100001100000102??0000?110?121010001001000011????00??11???1??0111001101110?01000101010210000100211?00010244011?000110111010111100201000000021?11001?111013?{1 2}01???1101??1?110021???1???11111101000100021???051??101{0 1}10110211101010002100{0 1}0100112001001?10214????11101101111?1?1210011?00?0?120003

Leptoropha_talanophora ??????????001?0?0??00011????0?2010210000010?0?0010000000011000?010110100000???101???10??1???20?020000210?0120103?0???????????????????0????0???????????????????????2????110????????????????????????????0?000??????????????????????????????????????????????????????????????????????????????0000????21???

Limnoscelis_paludis 31111000100011000000000000001111112101011?001000001???0001?000011110000000102110?01010011110000000200220?01010344011?000112111010110001211000000020?1110001000131011???10011?1?1100200011???011?110010011102111??????11??011102111000110021612010?10100110?010?1411111110110110?00?????1?1000111120004

Makowskia_laticephala 2101000011011100000000110000111010210000000?0100000010000100030110110001011001111210000?10011001100002??101201{0 1}3?010010011000101001110021100000?02??10??011201131211???1100??01110?????????????1??????1000020112?41101?1110100?11???01?00???????11??200?????1?1??????111?10???????1??????0000?00?21???

Megalocephalus_pachycephalus 100000100000011010000000010100100021000000000000001???101100001?000000000000?010230101001100120000210010200000033000010011201100101110?0010001???00010100000011010001011001110110002011101111100?101000000010????????????????????????????????????????????????????????????????????????????0000????00???

Microphon_exiguum 2?0100000?000100000000100000010010210000010?0?0000001000011000001011010101100?101?10100?11?0000020000210?01201233011?10000011101100110021?000????2??????0???001???2????110???0??10?2??????????????????0000020????????????????????????????????????????????????????????????????????????????0000????21???

Oedaleops_campi 31011?????001100000002??0000?1101121?1(0 1)1001?1101011????00??000?10??100100011?110?0??100?1?1??100{1 2}0201{0 1}?0?00000?4?1?????????????????????????????????????1????????????????????????????????????????????????1????????51?001?111?10210?01???002100{0 1}0200?120?????????????????????????????????????00???120???

Orobates_pabsti ??011??01100110000000000001011101121010111001100001???0101?000?11101000001000110?{1 2}1?100?1?00000010201020?110013440????1????101010110?122000000000?10??100012101?122?????101??1?11102????????111?11001?001?12210??2?????1?11?10211?00010012111101000?20?1??1010?1411111???1??110101?101110211111110?004

Ossinodus_pueri ???00?2???????0?0?????????0?0?1?00?0000000??0??0?00???00010000?1?00?????0?01????1??????00?0?2???????00??200???01??000001?0100000000100???1?0000?02??????????????1?????????????????????????????????????001?01?1010?111?0????????????????????1010?0100?01010?000???????????00??1??????????????0???0?????

Palaeoherpeton_decorum ??01????0?00100000000000100000101021000000?00?01000100000110010100000000000010101110001?1?0?11002000023?20100013?0????????1????????0?0?0110??00???1???????????????0???????????????????????????????????0?1?02??????????????????????????????????????????????????0??????????????????????????0000????00???

Paleothyris_acadiana 110100?0??001110000002??001011101121110001001?00101???010?0100010111000?01000110?01010001000110010200011101000{2 3}44011?00011211????????01201000000021011000011101?121????1101??1?1100???????????????????0010010???1????01??11110211111110001100011110120010011102111110111?0101101010??????1000101120103

Pederpes_finneyae {0 1}?0?0????????100000100?0000010?000????1000000?000001101011000011100000000?00???02??0101?010?210110?0????20000021?0?0010????010001000?0?0?10000000???????0010000??20??????0????000??1?????0???????0????0?1002?0011??011??210110100000010001010?0?0000210110?0012?30???10?0000010???1??????0000?0?0?0000

Petrolacosaurus_kansensis 110100?0??001100000002??00?001101121100?1100100?111???000??1000111000001?1102?10?01010001??0110010200211?01010?44011?10011?11101011000120100000002101110001110111211???1101??0111002????1???1111010001001001110214100011?111102111111110021000211001200100101011411101110?10110?01???????1?00101121113

Pholiderpeton_scutigerum ??0100?0??00?10?00?0???01011011?1??10??0001001010?00?00000100101?0??0000000010?001010000110011000020003?200000233011?0001110?10000000001010??0000200101000101013120000110011101100020101010101110122000000101101?1???0001??0?0{1 2}?0?0111??0214???????????????0102??????11110010101??1??????0000000?00??3

Proterogyrinus_pancheni ??????????????????????????????1?10?10000001?0??0?000?0?0??1001????1??????????????1??????????????????02??????0?14?0?????????????????????????????????????????????????????1?011?01100?00101????0100?122001???1????????00?????0?????1???????????????1011?????????????????1110010010??????????00?0????00???

Proterogyrinus_scheelei 100100?0??00001000000100?0100010102100000010010100000000011001010011000000000010011110?0110011002010023?200000234011??00???011001?0000?0010?00000?00101000101013110????10011?0?100?20?000101010??12200001010010?1100000111001020110001110114011010112001101010201000011000000100001??????0000000100003

Seymouria_baylorensis 31010000110011000000001000100110102100000001010000011000110003011010000101000110121000111110200000100210201000044010010011001101101110021100000002001000011200131211???1101110111002010101010111111101001002010?03?0011111111021110001100216120100?02001???00011401001110110110100?0001110000000121003

Seymouria_sanjuanensis 31?10??0110011000000001000?0011010210000000?010000011000110003011011000001000110121?10011?10000?001002??20100004?0????????????????????02?????????????????11????31?????????????????????????????????????0?1?02??0??5??????11111021110001100216?2?????????1100?0011401?0111?100110100?0001110000?00?2100?

Silvanerpeton_miripedes 21010??0??000100000001000000001010210000000?0?0000000000000001010001010000002010011?100?1?002100100002??100000{0 1 2}4?01001??11001???????10?00100?00002??10?000101010?20????1?01??01?00??????0???0100?1????0000010?0?110000????0?10?00001110002030{0 1}1?????2??1??0010{1 2}?{0 3}????1100000?1??001??????0000?00010002

Solenodonsaurus_janenschi ???1???0??0011?0000010010010?111112101110???0?01001???00111001??0011000001110?10?{1 2}1?100?1???1000010002???010??24?0??????????????????1?????????????????????????????0??????0???????0?2??????????????????0?0?01?1???310?0?????110211?00?110020????????????????0102?1????111001?010???????????000????{0 1}0???

Spinarerpeton_brevicephalum 210100001100110000000110000011201021000000100100000010000100030110110001001000111210100?10001101000002??20120103?0101100?10001010011100211?0000002?110?001120013121????1100??01110?2?????????????????11000010?12?21111?1100110?01?00010002?01?0111??2???????101?????????010????1??1??????0000????21???

Tseajaia_campi 21011?00??00110000001000001001100121110111000100001???0001000001100000000101001?021?100?10000000102010??11101034?01????01?210??????100221000000002?0101000020013110????1101??1?11002???10101011?11001?001002210?031?011???1?1021110?010002161201001120?1??0?1021411111110110110100????11?1100111100???

Utegenia_shpinari 210100000?001100000000010000011010210000010?0100000000000110020110110000000001100110100?1000210010100210101000231010010011001001110110021?000????2?0?0??011200121201???110???01110?201010101011001???11000020112?51001??{1 2}???1?????0??110?10?1{0 1}??????20?1???010103????0100000010?0?1??????0000000110003

Varanops_brevirostris 3101(0 1)0??0000?100000002??00??01101121??011010110?001????10??0000110110000101??110?00?10111110200000201120?01000?441????????????????????????????????101111??????????01???10011?1?1110211111???1111110000011002011??5000011?111102?11010100021010{0 2}1111120010011102141010111?01?11?1?1?????1?2?00101120114

Westlothiana_lizziae {1 2}?0100?00?1001?00?000120?010011011210000000?0?0??01???000??000?110110010100000?0??1?100010002?00000002???010003??0????????1?11011?1?10?00?0000000?1?????000?101?121?????10??????10????????????????????0000010????????0??011110211?0111000216001111012011001?10211????11101101110?????????0000?00100003

Whatcheeria_deltae {0 1}0000?{0 1}0??0001000000?000?0?0001100110000001001010001100011000{1 4}010000000101?0?0100???11??0??011111?2000102000003220?0?10????010001?0??0?0?0??0????2??????0??01013?200011100???00?0???010001101001?0000?011002000?051001?110011010000001?10?1100000??0?10101?111201????1011000010???0??????0000?00020??0

Eldeceeon_rolfei 10?10?{0 1}???0011000000011?000000101021?(0 1)00000?0?0000000000001001?100110100001020101{0 1}1?100?11001111102002??100001{0 1 2}4?01????0??1?110??1??1??011000000020?????00?????0?10????1101????????????????????????????10002011??21?0???????1??00?011100?2130{0 1}12????21010000101?4????1101000111?000??????0000?0012100{2 3}

Termonerpeton_makrydactylus ???????????????????????????????????????????????????????????????????????????????????????????????????????????????????????????????????????????????????????????????????????????????????????????????????????????????????????????????????????????5011??????0?0??0?10{1 2}??????1100000?11??00?????????????1??113

;

END;

============================================================================
